# Mental health trajectories before, during, and after the COVID-19 pandemic among patients attending a psychosomatic internal medicine clinic in Japan: A retrospective quantitative study

**DOI:** 10.1097/MD.0000000000051430

**Published:** 2026-11-06

**Authors:** Fumio Shaku, Motoko Ishiburo, Masako Miwa, Shuichiro Maruoka

**Affiliations:** a Nihon University Graduate School of Social and Cultural Studies, Tokyo, Japan; b Department of Psychosomatic Internal Medicine, Nihon University Itabashi Hospital, Tokyo, Japan.

**Keywords:** COVID-19, mental health, pandemic, psychosomatic, sleep disturbance

## Abstract

The coronavirus disease 2019 (COVID-19) pandemic has exacerbated anxiety and depression globally, particularly among individuals with mental health conditions and chronic illnesses. However, the extent to which these mental health effects have persisted following the pandemic remains unclear. This study examined changes in mental health before, during, and after the COVID-19 pandemic among first-time patients attending a psychosomatic internal medicine clinic in Japan. The legal reclassification of COVID-19 to “Class5” status under Japan’s Infectious Diseases Control Law, which took effect on May 8 2023, serves as the benchmark marking the beginning of the post-pandemic era, designated as the beginning of the post-pandemic period. The General Health Questionnaire (GHQ)-30, State-Trait Anxiety Inventory, and Center for Epidemiologic Studies Depression Scale (CES-D) were used to assess general mental health, state and trait anxiety, and depressive symptoms, respectively. Participants were classified into pre-pandemic (n = 87), during-pandemic (n = 322), and post-pandemic (< 1 year: n = 75 and 1–2 years: n = 68) groups. The Mann–Whitney *U* test was used to compare mental health outcomes across the study periods. A total of 552 participants completed the survey (237 men and 315 women; mean age: 44.57 ± 19.56 years). Within 1 year after the pandemic, GHQ total mean scores, sleep disturbance, anxiety and dysphoria, and CES-D mean scores were significantly higher than those in the pre-pandemic group (GHQ total: 16.32 vs 13.57, *P* = .015; sleep disturbance: 3.70 vs 2.65, *P* < .001; anxiety and dysphoria: 3.14 vs 2.35, *P* = .016; CES-D: 26.53 vs 21.39, *P* = .005). Sleep disturbance scores were also significantly higher within 1 year after the pandemic than during the pandemic (3.70 vs 3.19, *P* = .015) but were significantly lower in the 1 to 2 years post-pandemic group than in the within-one-year post-pandemic group (2.65 vs 3.70, *P* = .022). These findings suggest that symptoms of anxiety, depression, and sleep disturbance persisted during the early post-pandemic period but gradually improved over time. Particularly, sleep disturbance remained one of the most persistent mental health concerns, highlighting the importance of continued attention to the mental health of patients with psychosomatic disorders following the COVID-19 pandemic.

## 1. Introduction

The coronavirus disease 2019 (COVID-19) outbreak has been a severe stressor worldwide, as it falls outside the normal spectrum of human experience, poses a significant threat to life, and evokes a sense of powerlessness and profound anxiety.^[1]^

Global social distancing protocols enacted during the pandemic have profoundly reshaped the quality of life for millions of individuals. These measures have transformed established norms across education, professional environments, family dynamics, and general social interactions.^[2]^

Following the pandemic, Bourmistrova et al suggested that the global increase in reported mental health sequelae was more likely due to psychosocial factors rather than the direct long-term effects of the virus.^[3]^

For example, COVID-19 acted as a catalyst for a mental health crisis. According to meta-analyses, specific stressors such as financial instability and strain on healthcare systems led to increases of 20 to 30% in rates of depression and anxiety.^[4–6]^ Previous studies have also reported that emotional dysregulation, fatigue, and insomnia have become widespread and often co-occur with severe disorders.^[7,8]^ Although these problems are thought to have occurred worldwide, there are regional differences in mental health research.

Most mental health research continues to focus on Western, educated, industrialized, rich, and democratic populations, which limits the generalizability of findings to global majority communities.^[9–12]^

Conversely, somatic complaints (e.g., headache and gastrointestinal distress) are more commonly reported in Asian populations, whereas overt psychological symptoms tend to be emphasized in Western populations.^[13,14]^

Additionally, mental health is often influenced by individual background, and the pandemic is expected to have a greater impact on vulnerable populations.

For example, Fernández-Aranda et al online study conducted among patients with eating disorders found that 56.2% of the patients experienced increased anxiety, while 37.5% reported worsening symptoms.^[15]^ These findings suggest that individuals with stress-related and psychosomatic conditions may be more susceptible to the adverse effects of the COVID-19 pandemic than the general population.

Despite these established regional and clinical differences, empirical research on mental health during the pandemic’s later stages or after its formal conclusion remains scarce, particularly among non-Western clinical cohorts.^[16]^ In Japan, “Psychosomatic Internal Medicine” represents a unique medical specialty specifically dedicated to treating physical conditions that are significantly influenced by psychosocial factors. Because Japanese patients frequently present with somatized psychological distress rather than direct psychiatric complaints, psychosomatic clinics serve as a primary frontline clinical setting for detecting latent psychological vulnerability in Asian populations. Furthermore, alexithymia, which is a core characteristic of patients attending psychosomatic clinics, is marked by difficulty recognizing and verbalizing emotions, leading to unexpressed stress manifesting as chronic somatic distress.^[17,18]^ Consequently, such patients may be particularly sensitive to pandemic-related disruption, yet their psychological distress often remains unnoticed and understudied.

Therefore, a Japanese psychosomatic internal medicine clinic provides an ideal and essential framework to investigate how pandemic-induced psychological distress evolves into the post-pandemic period among a vulnerable population presenting complex mind-body interactions. To address this gap, this study specifically examines first-time visitors to a university hospital psychosomatic internal medicine service across pre-, during-, and post-pandemic periods. We hypothesized that patients presenting to a psychosomatic clinic during the early post-pandemic phase would exhibit persistent elevations in psychological distress and depressive symptoms compared to pre-pandemic baselines, followed by a gradual attenuation during the subsequent post-pandemic year.

## 2. Methods

This study employs a quantitative methodology to investigate the mental health status of individuals attending a psychosomatic internal medicine clinic. The analysis spanned 3 distinct periods: the pre-COVID-19 pandemic, during the pandemic, and following the reclassification of COVID-19 to Category 5 (where the government monitors the situation but does not impose strict, uniform restrictions on daily life) under the Infectious Diseases Act.

The study received ethical approval from the Human Subjects Institutional Review Board of the Nihon University Itabashi Hospital Clinical Research Judging Committee (Approval Number: RK-231212-10). All procedures adhered to the ethical standards outlined in the Declaration of Helsinki. Informed consent was waived and managed via a website-based “opt-out” process. Participants who opted out were deemed to be declining participation and were excluded from the final data analysis.

### 2.1. Participants and procedure

This retrospective study included patients who were first-time visitors to Nihon University Itabashi Hospital. Participants were categorized into 4 groups based on their initial visit date relative to the COVID-19 pandemic timeline (Table 1):

**Table 1:** Period covered by the survey.

Groups	Period covered by the survey
Group A	11/1/2018-12/31/2019
Group B	4/7/2020-5/6/2023
Group C-1	5/82023-5/7/2024
Group C-2	5/8/2024-4/25/2025

- Group A (before the pandemic): patients who visited between November 2018 and December 2019.
- Group B (during the pandemic): patients who visited between April 7, 2020 (coinciding with the first Japanese government-declared state of emergency), and May 6, 2023.
- Group C (after the pandemic): patients who visited from May 8, 2023, to April 25, 2025.
- Group C was further divided into Group C-1 (May 8, 2023–May 7, 2024) and Group C-2 (May 8, 2024–April 25, 2025).

The inclusion criterion required participants to complete psychological testing and be present for their first visit to the department (including external referrals). The exclusion criteria were as follows: inability to complete the psychological tests, formal request to withdraw from the study, and ineligibility as determined by the principal investigator. After excluding patients with incomplete data or previous clinic attendance, a final cohort of 552 patients was retained. Data were collected through retrospective analysis of anonymous questionnaires administered at the patients’ first visit. These included demographic details and completed routine psychological assessments: the General Health Questionnaire (GHQ-30), the State-Trait Anxiety Inventory (STAI), and the Center for Epidemiologic Studies Depression Scale (CES-D). This clinical study utilized only existing medical information and did not involve invasive procedures or interventions. Consequently, formal written informed consent was not mandated by governmental guidelines. Research information and the study’s purpose were publicly disclosed, allowing patients the opportunity to refuse participation (opt-out).

Information regarding individual participants’ history of severe acute respiratory syndrome coronavirus 2 infection, vaccination status, and post-viral sequelae (e.g., Long COVID) was not recorded as part of routine initial clinical screening, representing a procedural constraint of this retrospective design.

### 2.2. Psychometric measures

These measures were chosen because they are minimally invasive for outpatients, have gained trust domestically and internationally, and allow for a multifaceted assessment of mental health, including anxiety and depression.

#### 2.2.1. GHQ

The GHQ is a self-report screening tool for nonpsychotic psychiatric disorders,^[19]^ frequently employed in epidemiological surveys and clinical settings. We utilized the GHQ-30, a commonly used shortened version of the GHQ-60 developed by Hayasaka et al for the Japanese population.^[20]^ The GHQ-30 excludes somatic items, focusing on essential psychological distress, and is characterized by an equal balance of positively and negatively worded items with consistent response anchors.^[21]^

#### 2.2.2. STAI

Anxiety was assessed using the STAI, a self-report inventory comprising 2 distinct scales. The State Anxiety Scale measures anxiety responses relevant to a specific moment, while the Trait Anxiety Scale measures general, enduring anxiety levels.^[22]^ Scores range from 20 to 80, with little or no anxiety (<30), moderate anxiety (31–49), or extreme anxiety (≧50).^[23]^

#### 2.2.3. CES-D

Depressive symptoms were evaluated using the well-established, 20-item CES-D self-report scale. Total scores range from 0 to 60 and are interpreted as normal (0–15) or indicative of depression (16–60). The CES-D, like the GHQ-30 and STAI, is considered reliable for use within the Japanese population.^[24]^

### 2.3. Sample size

The sample size was determined using preliminary aggregated data collected before and after the declaration of the state of emergency. The calculation was based on the GHQ score, one of the primary study outcomes. According to the preliminary data, the mean GHQ score before the declaration of the state of emergency was 12.73 (standard deviation = 8.25), whereas the mean score after the declaration was 15.80 (standard deviation = 7.48). Based on these estimates, a minimum sample size of 300 participants was calculated to detect a statistically significant difference with 90% power at a 2-sided significance level of 5%.

### 2.4. Statistical analysis

All statistical analyses were conducted by a statistician affiliated with the Japan Institute of Statistical Technology (Tokyo, Japan) using SPSS Statistics 25 (International Business Machines Corporation). The score distributions for each questionnaire were examined using the Shapiro–Wilk normality test, and normality was rejected for all items. Therefore, nonparametric analysis was performed using the Mann–Whitney *U* test. These methods were employed to identify significant differences in mental health scale scores between the defined groups. Unadjusted 2-sided *P* values obtained from pairwise Mann–Whitney U tests were used in the primary exploratory analysis. To assess the robustness of the findings to multiplicity, the 5 pairwise comparisons for each questionnaire score were treated as 1 family of hypotheses, and Holm step-down procedure was additionally applied as a sensitivity analysis. Both unadjusted and Holm-adjusted *P* values are reported. In the primary exploratory analysis, an unadjusted *P* value of < .05 was regarded as providing nominal statistical evidence, whereas a Holm-adjusted *P* value of < .05 was considered to indicate that statistical significance was retained after adjustment for multiple comparisons. Effect sizes for pairwise comparisons were calculated as *r* = Z/√N, where Z represents the standardized test statistic from the Mann–Whitney *U* test.

## 3. Results

Of the 587 samples collected, 552 participants (94.0%) were included in the final analysis (237 men and 315 women; mean age: 44.57 ± 19.56 years). Participant distribution, descriptive statistics for psychological measures (GHQ, CES-D, and STAI), and group comparison details are summarized in Tables 2 – 4, respectively.

**Table 2:** Number of participants for each period.

Period classification	A: before the pandemic	87
	B: during the pandemic	322
	C: 0–1 year after the pandemic	75
	C: 1–2 years after the pandemic	68
Total		552

Within 1 year following the onset of the pandemic, participants exhibited significantly higher GHQ total, sleep disturbance, anxiety and dysphoria, and CES-D scores compared to those in the pre-pandemic period (*P* = .015, *P* < .001, *P* = .016, and *P* = .005, respectively). In comparisons regarding sleep disturbance across pandemic phases, scores peaked within 1 year post-pandemic, showing a significant increase compared to both the pre-pandemic and during-pandemic periods (*P* = .015). Subsequently, sleep disturbance scores significantly declined in the 1 to 2 years post-pandemic group compared to the within-one-year group (*P* = .022). By 1 to 2 years post-pandemic, sleep disturbance levels returned to baseline, showing no significant differences compared to either the pre-pandemic or during-pandemic groups. No significant group differences were observed in STAI scores across any period.

The corresponding effect sizes were moderate for the difference in sleep disturbance between the A and C-1 groups (*r* = −0.317) and small for the difference in CES-D scores between these groups (*r* = −0.226), with higher scores observed in the C-1 group. The effect sizes for the other nominally significant comparisons that did not retain statistical significance after Holm adjustment were small (|*r*| = 0.123–0.195) (Table 4).

**Table 4:** Unadjusted and Holm-adjusted *P* values for pairwise comparisons of questionnaire scores across study periods.

No.	Questionnaire score	Statistic	A vs C-1	A vs C-2	B vs C-1	B vs C-2	C-1 vs C-2
1	GHQ total	Unadjusted *P*	**.015**	.271	.358	.478	.198
		Holm-adjusted *P*	.073	.812	.812	.812	.792
		Effect size, *r*	−0.195	−0.090	−0.047	0.036	0.108
2	GHQA: General illness	Unadjusted *P*	.133	.345	.558	.803	.494
		Holm-adjusted *P*	.667	.000	1.000	1.000	1.000
		Effect size, *r*	−0.120	−0.077	−0.030	0.013	0.057
3	GHQB: Somatic symptoms	Unadjusted *P*	.677	.218	.864	.324	.342
		Holm-adjusted *P*	1.000	1.000	1.000	1.000	1.000
		Effect size, *r*	0.033	0.101	−0.009	0.050	0.080
4	GHQC: Sleep disturbance	Unadjusted *P*	**< .001**	.177	**.015**	.524	**.022**
		Holm-adjusted *P*	**< .001**	.354	.059	.524	.067
		Effect size, *r*	−0.317	−0.110	−0.123	0.032	0.192
5	GHQD: Social dysfunction	Unadjusted *P*	.187	.350	.751	.741	.868
		Holm-adjusted *P*	.937	1.000	1.000	1.000	1.000
		Effect size, *r*	−0.106	−0.076	0.016	0.017	0.014
6	GHQE: Anxiety and dysphoria	Unadjusted *P*	**.016**	.238	.500	.511	.313
		Holm-adjusted *P*	.079	.952	1.000	1.000	.952
		Effect size, *r*	−0.193	−0.096	−0.034	0.034	0.085
7	GHQF: Suicidal depression	Unadjusted *P*	.207	.977	.523	.335	.202
		Holm-adjusted *P*	1.000	1.000	1.000	1.000	1.000
		Effect size, *r*	−0.101	0.002	−0.032	0.049	0.107
8	CES-D	Unadjusted *P*	**.005**	.092	.405	.727	.369
		Holm-adjusted *P*	**.023**	.367	1.000	1.000	1.000
		Effect size, *r*	−0.226	−0.137	−0.042	0.018	0.075
9	STAI-T	Unadjusted *P*	.086	.200	.385	.728	.742
		Holm-adjusted *P*	0.431	0.800	1.000	1.000	1.000
		Effect size, *r*	−0.136	−0.103	−0.044	−0.018	0.028
10	STAI-S	Unadjusted *P*	.067	.163	.712	.818	.554
		Holm-adjusted *P*	.337	.653	1.000	1.000	1.000
		Effect size, *r*	−0.145	−0.112	−0.019	0.012	0.050

Values are 2-sided *P* values obtained from pairwise Mann–Whitney *U* tests. For each questionnaire score, the 5 pairwise comparisons were treated as 1 family of hypotheses. *P* values were adjusted separately within each outcome using Holm step-down procedure. Bold values indicate statistical significance after Holm adjustment at the 2-sided .05 level. The sign of *r* indicates the direction of the between-group difference according to the order of the groups shown in the table; a positive value indicates a higher score in the first-listed group, whereas a negative value indicates a higher score in the second-listed group.

GHQA: general illness, GHQB: somatic symptom, GHQC: sleep disturbance, GHQD: social dysfunction, GHQE: anxiety and dysphoria, GHQF: suicidal depression.

CES-D = Center for Epidemiologic Studies Depression Scale, GHQ = General Health Questionnaire, STAI = State-Trait Anxiety Inventory, STAI-T = Trait Anxiety Scale, STAI-S = State Anxiety Scale.

## 4. Discussion

Our hypothesis predicted that mental health issues would continue long after the COVID-19 pandemic, but the results of this study showed that some symptoms persisted for a long time, while others did not. The group assessed within 1 year after the pandemic showed significantly higher GHQ total scores (general mental health), sleep disturbance, anxiety and dysphoria, and depressive symptoms compared with the pre-pandemic group. In contrast, scores in the 1 to 2 years post-pandemic group were not significantly higher than pre-pandemic levels. These findings suggest that general psychological distress, sleep disturbance, anxiety and dysphoria, and depressive symptoms gradually diminished over time following the pandemic.

Even after the pandemic ended, sleep problems and symptoms related to anxiety and depression persisted. Even though the government’s infectious disease classification changed, mental health did not immediately return to pre-pandemic levels. However, 1 year after the classification changed to Category 5, mental health outcomes were no longer significantly different from those observed before the pandemic. One possible explanation is that, according to previous Japanese research, social, economic, political, and biological factors all contribute to mental and physical health disparities.^[25]^ Although not directly measured in the present study, ongoing economic burdens and altered social habits may serve as plausible contextual factors that potentially contributed to the persistence of psychological distress.

In this study, sleep disturbance appeared to be the outcome most susceptible to changes across the pre-, during-, and post-pandemic periods. Remarkably, sleep quality was worse within 1 year after moving to Category 5 than during the pandemic period. However, sleep disturbance scores were significantly lower in the 1 to –2 years post-pandemic group compared with the within-one-year post-pandemic group, suggesting gradual recovery over time.

One possible explanation is that the psychological effects associated with COVID-19 may have persisted even after the transition to Category 5. However, this study did not directly assess factors such as fear of COVID-19, pandemic-related anxiety, or risk perception; therefore, these possible explanations should be interpreted with caution. From a clinical perspective, sleep is a fundamental component of mental health and may serve as an early indicator of psychological distress. Even in individuals without clear anxiety or depression, poor sleep may contribute to the persistence of psychological symptoms.

COVID-19-related quarantine measures curtailed common coping mechanisms for stress, anxiety, and depression, such as regular exercise, socialization, and community engagement.^[26]^ Research into the psychological consequences of quarantine has identified diverse adverse effects, including confusion and resentment, fear of contagion, persistent frustration, and boredom, which may be exacerbated by prolonged isolation.^[27]^ In addition, media saturation and misinformation may have amplified fear and uncertainty, contributing to “pandemic fatigue” and a pervasive sense of helplessness.^[28,29]^ This psychological state may persist even after restrictions are lifted. Previous studies have suggested that the psychological effects of the pandemic may persist for several years.^[30,31]^ Another study also reported long-term effects of the COVID-19 pandemic among patients receiving treatment for psychosomatic disorders.^[17]^

The results of this study showed that the decline in sleep quality remains particularly pronounced even after the pandemic. Several systematic reviews and meta-analyses have revealed that sleep disturbances were common in different segments of the population during the COVID-19 pandemic.^[32–36]^ Moreover, some studies have reported increases in sleep duration (≥ 8/9 hours), as well as time spent in bed and napping habits.^[37]^

The most striking change that emerged from Ceolin et al study relates to the sleep–wake cycle.^[37]^ More than 40% of the participants experienced significant delays in both bedtime and wake-up time. Similarly, Marelli et al found significant delays in sleep timing, particularly among individuals with an evening chronotype during lockdown periods.^[38]^ Studies involving children revealed that they had longer sleep durations during the COVID-19 outbreak compared with pre-pandemic levels.^[39,40]^

In Japan, significant changes in sleeping habits were observed among children who had a later bedtime during school closures.^[41]^ Sleep disturbance has been associated with various adverse physical and psychological outcomes. Previous studies on delayed-onset disorders suggest that some conditions may emerge years after an initial traumatic event.^[42,43]^ In interpreting sleep disturbance, it is critical to recognize the bidirectional relationship between sleep disruption and affective symptoms; preexisting anxiety and depressive states disrupt sleep architecture, while impaired sleep concurrently exacerbates emotional dysregulation. Furthermore, future research should explore the potential relationship between Long COVID and mental well-being, including the mechanisms through which chronic physical symptoms may contribute to cognitive and emotional impairments.^[44,45]^ Continued post-pandemic research is warranted to better understand these long-term effects.

From a clinical perspective, alexithymia, a characteristic of psychosomatic disorders, limits patients’ ability to recognize or verbalize their symptoms.^[18]^ Alexithymia contributed to delayed recovery and the persistence of psychosocial symptoms following the COVID-19 pandemic. Individuals with alexithymic traits struggle to identify and express emotions, causing unaddressed stress to accumulate as chronic physiological arousal. This difficulty leads to somatization, where emotional distress is misinterpreted as physical illness or health anxiety. Furthermore, their reliance on avoidance-based coping mechanisms hinders effective emotional processing and stress management. Finally, impairments in emotional communication hamper social support-seeking, resulting in prolonged social isolation and sustained psychological distress. Therefore, healthcare professionals should be attentive to these difficulties and provide appropriate support.

### 4.1. Study limitations and directions for future research

This study has several limitations. First, it was conducted at a single university hospital, which may not accurately reflect the national context or the general population. Future studies should include multiple psychosomatic internal medicine clinics nationwide that implement the full range of selected psychological assessments to ensure broader data representation. Second, the study relied on self-reported, standardized questionnaires administered during routine clinical practice to minimize the burden on patients. Although instruments such as the GHQ-30 and the STAI are well-established and consistent with those used in previous research, they may have limited sensitivity to subtle contextual changes. Future research should adopt a more comprehensive approach to mental health assessment by incorporating additional quantitative measures and diverse assessment methods. We clarified that while evaluating Group B within a consolidated 3-year period provided a broader overview of the pandemic’s overall impact, it may have obscured temporal variations or short-term fluctuations tied to specific variant-driven waves (e.g., Alpha, Delta, and Omicron). We also noted that because our outpatient clinic operates on an appointment-only basis with waiting times often exceeding 1 month, granular time-series analysis of individual short-term waves was difficult to perform. Results that lost statistical significance after adjustment are signals to be validated in future studies.

Lack of data on long COVID status, we acknowledged that while long COVID symptoms are known to be associated with mental health, our study evaluated patients in routine clinical practice where universal COVID-19 testing was not feasible. Furthermore, given the presence of asymptomatic cases, it was difficult to definitively distinguish whether observed symptoms were attributable to long COVID or to other factors, including endogenous mental health conditions. Because responses to the individual items of each questionnaire were unavailable, Cronbach alpha coefficients could not be calculated, and the internal consistency of the questionnaire scores could not be directly evaluated in the present study sample. This limitation should be considered when interpreting the differences in questionnaire scores observed across the study periods. Finally, because of the cross-sectional nature of the data, the direction of causality remains unclear.

Future studies should also address potential confounding factors. Future analyses should incorporate appropriate statistical adjustments when examining associations with external variables, such as the presence of mental illness or other relevant characteristics. This may include the use of multivariate analyses (e.g., logistic regression) or adjustments for multiple comparisons, as appropriate.

## 5. Conclusion

This single-center observational study demonstrated significant temporal associations between pandemic policy phases and psychological distress among psychosomatic outpatients. Elevated levels of anxiety, depression, and sleep disturbance persisted in the early post-pandemic phase before showing significant resolution 1 to 2 years later. Clinical interventions in psychosomatic settings should focus on long-term sleep monitoring and addressing somatic manifestations of distress in post-pandemic care.

**Table 3:** Mean score and SD on the GHQ, CES-D, STAI.

Assessments	Group A			Group B			Group C-1			Group C-2		
	n	mean	SD	n	mean	SD	n	mean	SD	n	mean	SD
GHQ total	82	13.57	8.25	316	15.55	7.25	74	16.32	6.60	67	14.78	7.26
GHQA	82	2.79	1.69	317	3.10	1.54	74	3.23	1.49	68	3.09	1.47
GHQB	82	2.44	1.77	317	2.27	1.56	74	2.31	1.46	68	2.07	1.49
GHQC	82	2.65	1.70	317	3.19	1.62	74	3.70	1.39	68	3.03	1.74
GHQD	82	1.88	1.67	317	2.31	1.78	74	2.23	1.70	68	2.24	1.96
GHQE	82	2.35	1.93	317	2.96	1.74	74	3.14	1.65	68	2.76	1.89
GHQF	82	1.46	1.86	317	1.73	1.97	74	1.86	2.02	68	1.41	1.77
CES-D	83	21.39	12.47	317	25.24	12.55	74	26.35	11.44	68	24.56	12.34
STAI-T	86	52.53	13.53	315	54.56	12.34	74	56.00	11.79	68	55.13	12.88
STAI-S	86	51.74	11.81	316	54.41	11.62	74	54.70	11.98	68	54.01	11.06

GHQA: general illness, GHQB: somatic symptom, GHQC: sleep disturbance, GHQD: social dysfunction, GHQE: anxiety and dysphoria, GHQF: suicidal depression.

Group A: before the pandemic, Group B: during the pandemic, Group C-1: 0–1 year after the pandemic, Group C-2: 1–2 years after the pandemic.

CES-D = Center for Epidemiologic Studies Depression Scale, GHQ = General Health Questionnaire, SD = standard deviation, STAI = State-Trait Anxiety Inventory, STAI-T = Trait Anxiety Scale, STAI-S = State Anxiety Scale.

## Acknowledgments

The authors thank the Japan Institute of Statistical Technology (www.jiost.com) for performing the formal statistical analysis. We thank Editage (www.editage.com) for English language editing and journal submission support. The authors have authorized the submission of this manuscript through Editage.

## Author contributions

**Conceptualization:** Fumio Shaku, Motoko Ishiburo, Masako Miwa, Shuichiro Maruoka.

**Data curation:** Motoko Ishiburo, Masako Miwa.

**Formal analysis:** Fumio Shaku.

**Investigation:** Fumio Shaku, Motoko Ishiburo, Masako Miwa, Shuichiro Maruoka.

**Methodology:** Fumio Shaku, Motoko Ishiburo, Masako Miwa, Shuichiro Maruoka.

**Writing – review & editing:** Fumio Shaku.
